# Mantle fluids associated with crustal-scale faulting in a continental subduction setting, Taiwan

**DOI:** 10.1038/s41598-019-47070-2

**Published:** 2019-07-25

**Authors:** Ai-Ti Chen, Chuan-Chou Shen, Timothy B. Byrne, Yuji Sano, Naoto Takahata, Tsanyao Frank Yang, Yunshuen Wang

**Affiliations:** 1 High-Precision Mass Spectrometry and Environment Change Laboratory (HISPEC), Department of Geosciences, National Taiwan University, Taipei, 10617 Taiwan, ROC; 2 Research Center for Future Earth, National Taiwan University, Taipei, 10617 Taiwan, ROC; 3 Center for Integrative Geosciences, University of Connecticut, Storrs, CT 06269-1045 USA; 4 Atmosphere and Ocean Research Institute, The University of Tokyo, Kashiwanoha, Kashiwa, Chiba 277-8564 Japan; 5 Department of Geosciences, National Taiwan University, Taipei, 10617 Taiwan, ROC; 6 Central Geological Survey, MOEA, Taipei, 23568 Taiwan, ROC

**Keywords:** Geochemistry, Tectonics

## Abstract

We report noble gas signatures of groundwater, hot springs, and bedrock samples from a major fault system that separates regional-scale blocks of accreted, continental materials in southern Taiwan. Despite the continental setting, the isotopic signatures argue for the presence of mantle derived fluids, suggesting that the active fault system is deep-seated. This is consistent with deep, non-volcanic tremors identified in the same area. We speculate that the mantle fluids are escaping along a crustal-scale fault marked by clusters of non-volcanic tremors directly beneath the southern Central Range. The evidence of these tremors and electrical conductivity anomalies along the strike of the fault recognized previously correlated up dip with the surface trace of a major active fault support the hypothesis.

## Introduction

Convergent plate boundaries, where major earthquakes and catastrophic volcanic eruptions preferentially occur, are responsible for transferring significant volume of fluids into and out of the mantle^1,2^. Noble gases, especially helium isotopes (^3^He/^4^He) are natural tracers for tracking fluid migration process at crustal scale^3^ and circulation of fluids between mantle, crust, and atmosphere. With unique helium isotopic signatures^4^, and neon-helium ratios (^20^Ne/^4^He) of observed samples, important key to study the sources of geofluids can be deduced^2,5,6^. These ratios, integrated with geophysical data, can offer clues on the processes controlling regional-scale fluid transfer between the mantle and the crust. Along convergent plate boundaries, where oceanic crust is subducted, geophysical and geochemical studies suggest that fluid-rich rocks are subducted to mantle depths where they are dehydrated, hydrating the mantle and contributing to the production of arc magmas^7,8^. Helium isotopes of fluids escaping through major structures along these margins also suggest significant fluid transport of mantle-derived fluids to the surface^5^. In contrast, in areas where continental crust is being subducted, the role that mantle fluidization plays in the deformation and accretion of crustal materials is not well-known (e.g., Klemperer *et al*.^9^). The arc-continent collision in Taiwan represents one of the few places in the world where continental crust is actively subducting and, therefore, provides a rare opportunity to evaluate the role of mantle fluids in hydrating slabs of continental orogenic systems.

**Figure 1:**
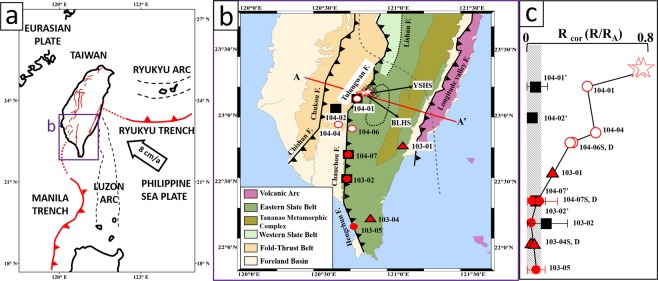
Sampling location and geographical distribution of helium anomalies. (**a**) Plate tectonic setting of Taiwan showing subduction of the Philippine Sea and Eurasian plates in the area of Taiwan^14^. (**b**) Tectonostratigraphic units, major faults and sample locations in southern Taiwan. Stars: hot spring sites; red circles: groundwater wells along the Tulungwan-Chaochou-Hengchun Fault system (TCH); red triangles: groundwater wells away from the TCH; square symbols: bed rock samples; Crosses (X) show area of crustal-scale (5–20 km) low resistivity zone^28^; black dotted line marks surface projection of non-volcanic tremors^19^. Grey dashed line shows the surface trace of slab deflection at 60 km depth16. (**c**) North-south distribution of Rcorvalues with 2-sigma uncertainties among sites. Numbers with S, D represent two samples were used in the same area at different depths (see Supplementary Table 1). Samples of rock are indicated with an apostrophe. With correction for atmospheric helium, Rcorvalues represent the contribution of crustal and mantle-derived components to the R/R_A_ values of samples, which is easier to differentiate the samples from atmospheric helium dominated ones. Note that some Rcorerror bars are smaller than the symbol size. Rcorvalues increase close to the area of tremor and the high conductivity zone.

The Taiwan orogenic belt is located at the plate boundary zone between the Eurasian and the Philippine Sea Plates and results from active subduction, accretion and exhumation of Eurasian continental crust along the Manila Trench^10–13^ (Fig. 1a). The subducted Eurasian lithosphere beneath Taiwan dips 60° east in southern Taiwan but steepens to nearly 90° in northern Taiwan^14^. This change in dip of the subducting plate is manifested by a relatively sharp deflection^15^ in the NW-SE striking structures of the lithosphere beneath central Taiwan^16^ (Fig. 1). The continental margin in the subducting plate also trends northeast, highly oblique to the trend of the orogen, and projects eastward to the core of the collisional orogen in Taiwan. As a result, the transition from relatively thin continental crust to thicker crust occurs beneath central Taiwan.

The orogenic belt of Taiwan (Fig. 1b) is composed by a Late Miocene fold-and-thrust belt, a slate belt of Eocene to middle Miocene age and the Tananao Metamorphic Complex of pre-Eocene age. In the southern Central Range (Fig. 1b), the slate belt is composed of the middle Miocene Changshan Formation and the late Eocene Pilushan Formation which includes local outcrops of conglomerate and volcanic layers^17^. Tananao Metamorphic Complex crops out east of the slate belt and contains late Mesozoic intrusive rocks and shallow water carbonates considered to be Late Paleozoic in age^13,18^. The boundary between the slate and fold-and-thrust belt is marked by a major, east dipping system of thrust faults, the Tulungwan-Chauchou-Hengchun Fault system (TCH). The TCH is represented by three fault segments with different trends. In the north, the Tulungwan Fault strikes northeast and separates the slate and fold-and-thrust belt. Further south the boundary strikes north-south and is marked by the Chauchou Fault. Finally, in the southern Central Range, the boundary trends northwest and is marked by the Hengchun Fault (Fig. 1b). Huang and Byrne^18^ compiled geological, structural, and geodetic data along the Tulungwan and northern Chaochou segments and proposed that the fault system is active and driving uplift in this area.

In the core of the Central Range, east of the TCH, Chuang *et al*.^19^ also identified a cluster of ambient tremors at 20 to 40 km depth. Ide *et al*.^20^ stacked a subset of the tremors and proposed a thrust fault focal mechanism as previously suggested by Tang *et al*.^21^. The cluster is overlain by swarms of earthquakes along normal faults, delineating sub-vertical, planar clusters that correlate temporally with the tremor activity^19^. This crustal scale zone of seismic activity therefore records a vertical gradient in crustal stress from thrust faulting in the deep crust to extension at shallower crustal levels^22^.

Bertrand *et al*.^23^ collected electro-magnetic data across the southern Central Range and recognized a zone of anomalously low resistivity (<30 Ωm) at the northern end of the Tulungwan Fault segment (Fig. 1b). The mapped resistivity values suggest 1–2% of melting generated in the lower crust^23^. Based on these observations, Chuang *et al*.^19^ argued that metamorphic dehydration and fluid-flow were critical drivers in generating the tremors and earthquake swarms within this part of the orogen.

To understand the role of fluids in the arc-continent collision system, we investigated the TCH using helium isotopes (^3^He/^4^He) and ^20^Ne/^4^He ratios, as the fault system appears to represent a major, and possibly crustal-scale, structure in the orogen. Indeed, previous studies on gas exhalations in Taiwan^24–26^ revealed multiple helium sources. Yang *et al*.^24–26^ identified mantle-derived helium in the fold the and thrust belt and related exhalation to mud volcanoes associated with a subsurface fault zone, although no specific structure was identified. Recently, Sano *et al*.^27^ measured helium isotopic ratios along the convergent plate boundary of the western Pacific Plate and suggested that mantle-derived helium in southern Taiwan was related to collision tectonics with some magmatic heat and active faults, although no specific structure was identified. In this study, we collected groundwater and hot spring samples along the surface track of the TCH and bedrock and groundwater samples away from the TCH for reference. The aim was to identify mantle-derived helium and understand its relation to anomalous seismic tremors^19^ and electrical resistivity^28^.

## Results

Fluids sampled on the Earth’s surface might contain helium derived from three different sources: (1) the mantle; (2) the crust; or (3) the atmosphere. Convective mantle as sampled at the mid-ocean ridges contributes primordial ^3^He, generating high ^3^He/^4^He (R) ratios times the atmospheric ratio^6^, e.g. deep plume-related mantle reservoirs are characterized by higher than 8 R/R_A_^29^. Subcontinental lithospheric mantle shows typical R/R_A_ values of 6.3 ± 0.4^30^. Both mantle sources have ^20^Ne/^4^He values ≪0.01. Continental or oceanic crust generates ^4^He from the decay of U and Th, and ^3^He from thermal neutron capture of ^6^Li with typical R/R_A_ ratios of 0.02–0.05^31^ with ^20^Ne/^4^He values ≪0.01^32^. Helium in the atmosphere is dissolved at solubility equilibrium (air-saturated water or ASW) with R/R_A_ ratios of 0.983^33^ and ^20^Ne/^4^He ratios of ≈3.7 at 25 °C^4,34,35^.

^4^He and ^20^Ne concentrations, ^20^Ne/^4^He and ^3^He/^4^He ratios measured in hot springs, groundwater and in rocks in the southern Central Range are listed in Supplementary Table 1. In rock samples, ^4^He concentrations range from 6.8 × 10^−10^ to 4.6 × 10^−9^ cm^3^/g and the ^3^He/^4^He ratios have a relatively narrow range from 0.05 to 0.13 R_A_. The ^20^Ne/^4^He ratios are relatively low, <0.01. The ^4^He concentration in groundwater ranges from 6.2 × 10^−8^ to 6.2 × 10^−6^ cm^3^/g at sampling temperatures of 23.9–36.7 °C and 1 atm. Most water samples have much higher ^4^He content than expected at ASW (6.1 × 10^−8^ cm^3^/g at 25 °C and 1 atm)^6^ except for 104–07S and 103–05. ^3^He/^4^He ratios in the water samples vary significantly from 0.05 to 0.97 R_A_. ^20^Ne concentration in groundwater ranges from 3.4 × 10^−8^ to 2.4 × 10^−7^ cm^3^/g, and ^20^Ne/^4^He varies from 0.01 to 2.94.

## Discussion

**Figure 2:**
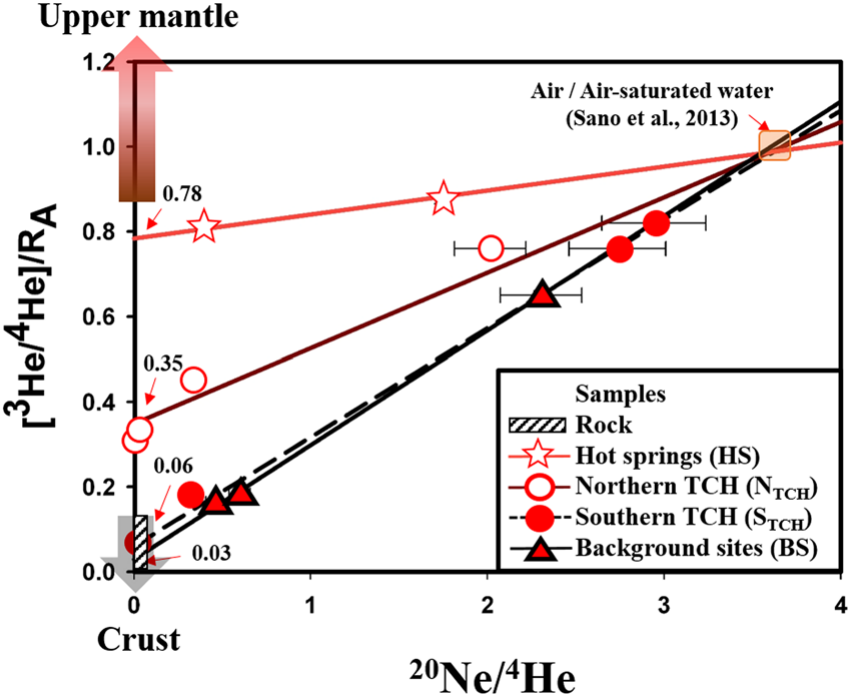
Plot of ^20^Ne/^4^He andR/R_A_ values for samples from the southern Central Range. The lines show proposed mixing curves of three end-member components: upper mantle, crust, and air/air saturated water with the vertical axis showing the contribution of ^3^He/^4^He from the crust or upper mantle. Error bar of R/R_A_ value is ±0.02 (2σ), smaller than the symbol size. The correlation coefficients (R^2^) of the four regression lines are 0.995 (HS, 3 points), 0.977 (NTCH, 5 points), 0.998 (STCH, 5 points), and 0.995 (BS, 4 points). TCH: Tulungwan-Chaochou-Hengchun Fault system.

Figure 2 shows the R/R_A_ values versus ^20^Ne/^4^He ratios in the southern Central Range. Groundwater sampled along the Hengchun and southern Chaochou Faults shows values falling on a mixing line between air and the crust. The mixing line intercepts the R/R_A_ axis at values of 0.03 and 0.06 R/R_A_, consistent with R/R_A_ values for bedrock samples 0.05–0.13 (see Supplementary Table 1 and methods for details) thus confirming the dominant occurrence of crustal helium in these samples, which are considered as “background sites”. Groundwater samples of 104–07 and 103-05 are observed to contain helium concentrations similar to ASW as they are derived from the “background sites” and shallow depth; thus, no influence from crustal source is expected. A second subset of the samples from the southern Central Range defines a second mixing line that intercepts the y-axis at 0.35 R_A_. These are groundwater samples exclusively from the northern Chaochou Fault and southern Tulungwan Fault. The value of 0.35 R_A_ is higher than values from the “crustal” mixing line, suggesting the addition of primordial ^3^He. The final two samples are from hot springs along the northern Tulungwan Fault and define a third mixing line with y-axis intercept of 0.78 R_A_. This value also reveals a significant addition of primordial ^3^He. All of these sites that define the third mixing line are located near the areas of non-volcanic tremors, high conductivity, an anomalous deflection in the subducted lithosphere and outcrops of volcanic rocks (Fig. 1b,c). The possible significance of these observations are discussed in the following paragraphs.

Outcrops of volcanic rocks in the Pilushan Formation near the TCH suggest a possible source of the mantle-derived helium with high ^3^He/^4^He ratios compared to the background sites. The extent of the volcanic rocks, however, is very limited^17^ and maximum degassing probably occurred when the rocks were extruded approximately 26 Ma, the age of the sediments associated with the volcanic rocks. Measurements of helium concentrations in olivine (10^−8^–10^−9^ cm^3^STP/g) are a hundred to a thousand times lower than in groundwater^7^. Considering the age of volcanic layers and the mineral contribution, very little ^3^He would therefore be available for contaminating groundwater. The limited extent of the volcanic layers also suggests that crustal radiogenic helium, ^4^He would dominate the helium signal of groundwater^36^.

**Figure 3:**
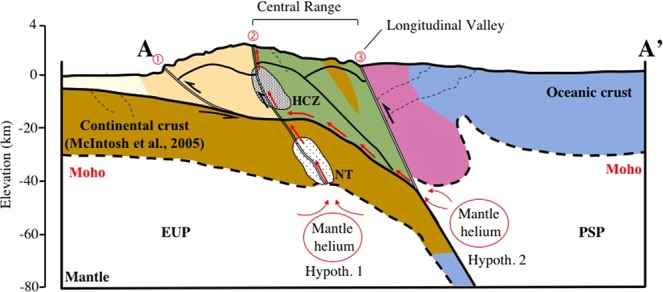
Schematic profile across line A-A’ in Fig. 1. Tectonic units are the same as in Fig. 1. Fault systems: ① is the Chishan-Chukou Fault, ② is theTulungwan-Chaochou-Hengchun Fault system (TCH), and ③ is the Longitude Valley Fault^18,54^. Dashed lines are the Moho surface^55^. Two shaded zones are the high conductivity zone (HCZ) and non-volcanic tremors (NT)^19,23^. Arc-continent collision in eastern Taiwan caused crustal thickening of the Central Range, exposing the Tananao Metamorphic Complex. Two hypotheses that explain the source of the mantle-derived helium are illustrated along this section: 1) fluids from Eurasian Plate (EUP) mantle travel along a crustal-scale shear zone marked, in part, by the tremor zone (NT) and 2) fluids from Philippine Sea Plate (PSP) mantle travel along the subduction zone boundary. In both hypotheses mantle derived fluids migrate to the surface along the northern TCH or Tulungwan Fault.

Three possible regional-scale tectonic processes or some combination of them are proposed to explain the transport of mantle-derived fluids sampled along the trace of the TCH. First, along-strike steepening and deflection of subducted Eurasian crust beneath the central part of the orogen^16^ may have concentrated strain and deformation, leading to failure and formation of a shear zone in the deflected crust. One possibility is that deflection of the crust was accommodated by the formation of a steeply dipping tear fault striking northwest, approximately parallel to the deflection. This tectonic feature could expose Eurasian mantle and if it penetrated the continental crust, may have acted as a conduit for mantle-derived fluids. Second, fluids associated with the exhumation of high-pressure mafic schists and metamorphosed peridotite exposed in the eastern Central Range may have escaped along the subducting plate boundary. Recently, several studies^37–39^ have proposed that the high-pressure metamorphic rocks exposed along the boundary between the magmatic arc and the accreted continental blocks in Taiwan were exhumed from approximately 50 km depth in the last 5 Ma. These exhuming rocks probably carried fluids that may have escaped to the surface along the east-dipping subduction zone (Fig. 3). In this scenario, the fluids and helium would have originated in the Philippine Sea lithosphere, rather than the Eurasian lithosphere. Finally, the third tectonic process may have been progressive imbrication of continental crust along a crustal-scale shear zone (see e.g., Chen *et al*.^22^). Resistance along the plate boundary may have increased as thicker continental crust is carried into the subduction zone. At the same time, prograde metamorphism in the subducted lower crust may have weakened the crust, leading to localized deformation, failure and development of a crustal-scale shear zone. This crustal-scale zone could then serve as a conduit for channelized flow to, and from the Eurasian lithosphere.

At the current stage, we are not able to distinguish these different scenarios. The occurrence of the tremors south of the trace of the deflection in the subducting crust, rather than directly above the deflection suggests that the deflection is probably not responsible for the escaping fluids. The north-south trend of the cluster of seismic tremors, which parallels to the TCH, suggests that imbrication of the continental crust along a crustal-scale shear zone, represented at the surface by the TCH, is more consistent with the observations.

Previous studies on the behavior of helium in active tectonic regions can help in understanding the source of the helium and mechanism of fluid transport^4,35,40^. In subduction zones, for example, the principle carriers of mantle helium are considered to be fluids degassing directly from mantle melts and traveling through the crust, possibly by buoyancy. In Taiwan, the active volcanic complex, the Tatun Volcano Group, is located several hundred kilometers north of the southern Central Range and is, therefore most likely not the source^41^. The Luzon magmatic arc, whose remnants crop out only 50–60 km east of the Central Range has been inactive for at least several Ma, making this arc also an unlikely source. The alternate scenario, i.e. degassing of fluids directly from mantle melts at depth, is proposed to explain helium anomalies in non-volcanic, tectonic environments, like the San Andreas Fault where mantle fluids are interpreted to pass through the ductile lower crust along a deep fault system^1,42^. Studies of helium isotopes associated with the North Anatolian and Karakoram Fault zones have also documented mantle volatiles, although no recent volcanism has been reported^9^. Recently, Sano *et al*.^43^ found an influx of mantle fluids along the Futagawa-Hinagu Fault zone located in Kumamoto earthquake region in Japan where no active magmatism is present. Sano *et al*.^43^ argued, however, that a small portion of mantle melt was present at depth and that the helium-bearing fluid was diluted by radiogenic helium as it traversed the crust via a permeable fault plane. More recently, Caracausi and Sulli^44^ discussed He data in a convergent continental setting and interpreted the possible occurrence of mantle melts in a continental setting as due to the delamination processes that allow the suction of the mantle wedge. We propose a similar interpretation for the helium anomalies in Taiwan. Although, there may not have been sufficient time or inappropriate conditions (i.e., stress regime) for the magma itself to reach the surface.

Support for the proposed deep mantle source comes from the spatial correlation of helium anomaly and the deep crustal tremors in south-central Taiwan. For example, McCrory *et al*.^35^ recently documented a similar spatial correlation between a significant component of mantle-derived helium and tectonic tremors in the Cascadia forearc. Matsumoto *et al*.^45^ confirmed that the fluids derived from fore-arc slab dehydration might be linked to the occurrence of long-period tremors in the Kii Peninsula, southwest Japan. Dogan *et al*.^46^ further supported this interpretation and argued that fluids liberated from the slab in the forearc region cause deep tremors and fracturing within the crust, and a fault system can provide an efficient path for the transfer of helium from mantle. In these areas the authors linked tectonic tremors with deep fluid transport from upper mantle and proposed that the tremors represented deep fractures that allow helium movement through upper mantle and crust.

## Conclusions

This study provides compelling evidence for an active, crustal-scale fracture zone in subducting continental crust in the core of the southern Central Range, Taiwan. The correlation between high helium isotopic ratios in groundwater and hot springs and non-volcanic tremors and a zone of high conductivity suggests that the tremors represent the presence of deep flow of mantle fluids, possibly along a developing fault or fracture zone in the subducted crust. Our results are consistent with previous studies suggesting that the TCH is active and accommodating imbrication of the subducted crust^18^. The new isotopic data, with a significant mantle signal, also argue for detachment of the crust from the mantle and the possible existence of melt in the lower crust and upper mantle.

## Methods

### Sampling locations and analyses

Hot spring and groundwater samples were collected from sites relatively close to and far away from the TCH (3–10 km and 30–50 km, respectively) (Fig. 1). Samples were collected using a copper tube with an inner volume of 15 cm^3^ sealed by stainless-steel clamps to avoid possible gas exchange of atmospheric helium^47^. Hot spring waters are considered to be more representative of deep-seated fluids due to their high-temperature conditions. Two natural hot springs along the northern TCH were sampled for this study: Yui-Suei (YSHS) and Bo-Lai (BLHS) (Fig. 1b, Supplementary Table 1). Eleven groundwater samples were collected from wells with water head depths of 10–100 m and elevations of 96–931 m above sea level along the northern (104-01, 104-04, 104-06), southern TCH (the rest ones), and at background sites away from the TCH (103-01, 103-04) (Fig. 1b, Supplementary Table 1).

Dissolved gases were carefully extracted from water sample to avoid air contamination, and introduced into an all-metal vacuum system^47^. Helium and neon were purified in activated-charcoal traps at liquid nitrogen temperature, together with titanium getters^48^. ^20^Ne/^4^He ratio was determined on a Pfeiffer QMS 100 quadrupole mass spectrometer, at the Atmosphere Ocean Research Institute, University of Tokyo, with a reproducibility of ±6% (2 RSD)^8^. After separating helium from neon using a cryogenic charcoal trap at 40 K^8,49^, helium isotopic ratios (^3^He/^4^He) were analyzed using a noble gas mass spectrometer, GV Instrument Helix SFT. Replicate measurements of the Helium standard of Japan^50^, show a reproducibility of ±0.03% (2 RSD)^49^.

Four rock samples, 100 g each, were collected from drilled well cores (Supplementary Table 1) at 90–100 m depth. The compositions of rock samples are metasandstones and fine-grained shales. Every subsample, 1–2 g, was put into a stainless-steel container containing a stainless-steel ball with a vacuum valve. The container with a stainless-steel tube is directly connected to the purification line under the vacuum. The crusher was evacuated overnight using a turbo molecular pump. Crushing method was adopted instead of conventional stepwise heating method to extract helium to minimize the contamination from the air and/or organic materials sticking on the grain surface of the rock samples^51^. Subsamples were shaken by hand for five minutes to release inclusive gases, which were then analyzed for isotopic compositions of helium and neon using the same techniques as water samples.

### Geochemical analysis

The helium isotopic composition in water samples can be altered by ASW originally trapped inside the aquifer. The resultant ratios are often corrected for potential ASW contamination. In this study, the correction is necessary for showing the geographical relationship of mantle-derived helium and tectonic setting. therefore, the contamination was corrected using the following formula^4,35,47,52^:

$${{\rm{R}}}_{{\rm{cor}}}=[{({}^{3}{\rm{He}}/{}^{4}{\rm{He}})}_{{\rm{meas}}}\mbox{--}{\rm{r}}]/(1\mbox{--}{\rm{r}});$$

$${\rm{r}}={({}^{4}{\rm{He}}/{}^{20}{\rm{Ne}})}_{{\rm{ASW}}}/{({}^{4}{\rm{He}}/{}^{20}{\rm{Ne}})}_{{\rm{meas}}};$$

where R_cor_ and (^3^He/^4^He)_meas_ denotes the corrected and measured ^3^He/^4^He ratios relative to air value. (^4^He/^20^Ne)_ASW_ is ^4^He/^20^Ne ratio of the ASW and (^4^He/^20^Ne)_meas_ is observed sample ^4^He/^20^Ne ratio. The (^4^He/^20^Ne)_ASW_ ratio was calculated by the solubility formula (^4^He/^20^Ne = 0.266–0.286 at 19–36 °C) and sampling temperature^6,32,53^. Uncertainty evaluation for calculated isotopic ratios followed the methods described by Sano *et al*.^47^.

## Supplementary information

Supplementary Table 1
