# Supplementary material for: Mantle fluids associated with crustal-scale faulting in a continental subduction setting, Taiwan
Source: Sci Rep. 2019 Jul 25;9:10805. doi: 10.1038/s41598-019-47070-2 (PMC6658536; doi:10.1038/s41598-019-47070-2)
Supplement: Supplementary file 1 — Supplementary Table 1 [file 41598_2019_47070_MOESM1_ESM.docx]

SUPPLEMENTARY INFORMATION

**Mantle fluids associated with crustal-scale faulting in a continental subduction setting, Taiwan**

*Ai-Ti Chen^1^, Chuan-Chou Shen^1,2^*, Timothy B. Byrne^3^*, Yuji Sano^4^, Naoto Takahata^4^, Tsanyao Frank Yang^5^, Yunshuen Wang^6^*

*To whom correspondence should be addressed:

Chuan-Chou Shen: Tel: 886-2-33665878; Fax: 886-2-33651917; email: [river@ntu.edu.tw](mailto:river@ntu.edu.tw)

&

Timothy B. Byrne: Tel: 860-416-6132; Fax: 860-486-1383; E-mail: [tim.byrne@uconn.edu](mailto:tim.byrne@uconn.edu)

**Supplementary Table 1. Information related to sampling locations and conditions, as well as observed ^4^He and ^20^Ne concentrations, helium isotopes, ^20^Ne/^4^He ratios in springs, ground water and rock samples^#^.**

| Sample name | Sample type | Sample location^#^ | Latitude | Longitude | Altitude | Depth | Water temperature | Lithology | ^4^He | ^20^Ne | ^3^He/^4^He | ^20^Ne/^4^He^a^ | r | Rcor |
| --- | --- | --- | --- | --- | --- | --- | --- | --- | --- | --- | --- | --- | --- | --- |
|  |  |  |  |  | m | m | ℃ |  | c.c.STP/g | c.c.STP/g | R/Ra (± 2σ) |  |  | R/Ra (± 2σ) |
| BLHS | spring | 1 | 120.71 | 23.11 | 473 | 0 | 55 | Sandstone | 1.08×10^-7^ | 1.89×10^-7^ | 0.87±0.02 | 1.74 | 0.54 | 0.73±0.06 |
| YSHS |  | 1 | 120.79 | 23.18 | 470 | 0 | 35 |  | 1.72×10^-7^ | 6.71×10^-8^ | 0.81±0.02 | 0.39 | 0.11 | 0.79±0.02 |
| 104-01 | Groundwater | 1 | 120.72 | 23.13 | 481 | 100 | 29.3 | Sandstone | 1.03×10^-7^ | 3.41×10^-8^ | 0.45±0.02 | 0.33 | 0.09 | 0.39±0.02 |
| 104-04 |  | 1 | 120.60 | 22.93 | 113 | 35 | 25 |  | 9.89×10^-8^ | 1.99×10^-7^ | 0.76±0.02 | 2.02 | 0.55 | 0.46±0.06 |
| 104-06S |  | 1 | 120.68 | 22.90 | 210 | 38 | 26.9 | Meta-sandstone | 3.84×10^-6^ | 1.07×10^-7^ | 0.33±0.02 | 0.03 | 0.01 | 0.32±0.02 |
| 104-06D |  | 1 | 120.68 | 22.90 | 210 | 100 | 25.6 |  | 2.27×10^-6^ | 1.80×10^-8^ | 0.31±0.02 | 0.01 | 0.00 | 0.31±0.02 |
| 104-07S | Groundwater | 2 | 120.67 | 22.71 | 150 | 32 | 23.9 | Meta-sandstone | 6.16×10^-8^ | 1.81×10^-7^ | 0.82±0.02 | 2.94 | 0.80 | 0.10±0.10 |
| 104-07D |  | 2 | 120.67 | 22.71 | 150 | 100 | 26.2 |  | 6.19×10^-6^ | 9.39×10^-8^ | 0.07±0.02 | 0.02 | 0.00 | 0.07±0.00 |
| 103-02 |  | 2 | 120.66 | 22.53 | 124 | 100 | 26.3 |  | 7.65×10^-7^ | 2.41×10^-7^ | 0.18±0.02 | 0.32 | 0.09 | 0.10±0.02 |
| 103-01 | Groundwater | 3 | 121.04 | 22.76 | 154 | 100 | 26.2 | Slate | 1.06×10^-7^ | 2.44×10^-7^ | 0.65±0.02 | 2.30 | 0.57 | 0.19±0.02 |
| 103-04S |  | 3 | 120.81 | 22.22 | 202 | 45 | 36.6 | Meta-sandstone | 3.79×10^-7^ | 1.72×10^-7^ | 0.16±0.02 | 0.45 | 0.13 | 0.04±0.02 |
| 103-04D |  | 3 | 120.81 | 22.22 | 202 | 100 | 36.7 |  | 3.30×10^-7^ | 1.97×10^-7^ | 0.18±0.02 | 0.60 | 0.17 | 0.01±0.02 |
| 103-05 |  | 3 | 120.71 | 22.16 | 96 | 40 | 25 | Sandstone | 7.61×10^-8^ | 2.08×10^-7^ | 0.76±0.02 | 2.74 | 0.75 | 0.05±0.08 |
| 104-01R | Rock | 1 | 120.72 | 23.13 | 481 | 100 | -- | Sandstone | 1.88×10^-9^ | ND | 0.06±0.08 | 0.00 | -- | -- |
| 104-02R |  | 1 | 120.58 | 23.05 | 353 |  | -- |  | 4.56×10^-9^ | ND | 0.05±0.04 | 0.00 | -- | -- |
| 103-02R |  | 2 | 120.66 | 22.53 | 124 |  | -- | Shale | 6.84×10^-10^ | ND | 0.13±0.14 | 0.00 | -- | -- |
| 104-07R |  | 2 | 120.67 | 22.71 | 150 |  | -- | Meta-sandstone | 1.26×10^-9^ | ND | 0.06±0.06 | 0.00 | -- | -- |
| ^#^Sample location: 1. near northern Tulunwan-Chaochou-Henchun Fault system (Chauchou Fault System, CFS), 2. near southern CFS, and 3. reference sites. Reported uncertainties for ^4^He and ^20^Ne concentrations are estimated at ±6% (2σ). Analytical uncertainties for each measurement is <0.5%. | | | | | | | | | | | | | | |
